# Evaluation of predictive models for delayed graft function of deceased kidney transplantation

**DOI:** 10.18632/oncotarget.22711

**Published:** 2017-11-27

**Authors:** Huanxi Zhang, Linli Zheng, Shuhang Qin, Longshan Liu, Xiaopeng Yuan, Qian Fu, Jun Li, Ronghai Deng, Suxiong Deng, Fangchao Yu, Xiaoshun He, Changxi Wang

**Affiliations:** ^1^ Organ Transplant Center, The First Affiliated Hospital, Sun Yat-Sen University, Guangzhou 510080, China; ^2^ Zhongshan School of Medicine, Sun Yat-Sen University, Guangzhou 510080, China; ^3^ Guangdong Provincial Key Laboratory on Organ Donation and Transplant Immunology, Guangzhou 510080, China

**Keywords:** delayed graft function, graft survival, deceased kidney transplantation, prediction models

## Abstract

**Background:**

This study aimed to evaluate the predictive power of five available delayed graft function (DGF)-prediction models for kidney transplants in the Chinese population.

**Results:**

Among the five models, the Irish 2010 model scored the best in performance for the Chinese population. Irish 2010 model had an area under the receiver operating characteristic (ROC) curve of 0.737. Hosmer-Lemeshow goodness-of-fit test showed that the Irish 2010 model had a strong correlation between the calculated DGF risk and the observed DGF incidence (*p* = 0.887). When Irish 2010 model was used in the clinic, the optimal upper cut-off was set to 0.5 with the best positive likelihood ratio, while the lower cut-off was set to 0.1 with the best negative likelihood ratio. In the subgroup of donor aged ≤ 5, the observed DGF incidence was significantly higher than the calculated DGF risk by Irish 2010 model (27% vs. 9%).

**Materials and Methods:**

A total of 711 renal transplant cases using deceased donors from China Donation after Citizen's Death Program at our center between February 2007 and August 2016 were included in the analysis using the five predictive models (Irish 2010, Irish 2003, Chaphal 2014, Zaza 2015, Jeldres 2009).

**Conclusions:**

Irish 2010 model has the best predictive power for DGF risk in Chinese population among the five models. However, it may not be suitable for allograft recipients whose donor aged ≤ 5-year-old.

## INTRODUCTION

Kidney transplantation has become a routine procedure in the treatment of patients with irreversible kidney failure [1]. Delayed graft function (DGF) is a complication after kidney transplantation and is a manifestation of acute kidney injury commonly defined as requiring dialysis within the first week post-transplantation [2]. The reported incidence of DGF in deceased donors has increased over the past decades [3], and is expected to rise further due to the use of expanded criteria donors (ECD) and donation after cardiac death (DCD) who show higher DGF rate than donation after brain death (DBD) [4–6]. Over the last three decades, the reported incidences of DGF in adult recipients from deceased donor kidney transplantation range from 15 to 30% [7].

DGF is a major obstacle for successful transplantation as it causes post-transplantation oliguria, increased allograft immunogenicity and risk of acute rejection episodes, and decreased long-term survival [8]. Due to the adverse influences of DGF on the transplantation outcome, significant efforts have been made to identify risk factors for DGF [7, 9–11]. Identification of risk factors for DGF is helpful for early preventive management. In addition, in attempting to develop an easy method to predict DGF, several clinical algorithms have been proposed and are reported to have good predictive power for DGF [12–16]. In 2003, Irish *et al*. have developed a multivariable logistic regression model including 16 variables of donors and recipients at the time of transplantation to predict the likelihood of DGF occurrence using the data on 13,846 cases of deceased donor kidney transplantation from the United States Renal Data System registry [14]. In 2010, Irish *et al*. further refined their predictive model to include warm ischemia time (WIT, total 17 variables) using the data from 24,337 cases of deceased donor kidney transplantation, which has a diagnostic accuracy (area under the receiver-operating characteristic curve, [AUC]) of 0.70 [13]. In 2009, Jeldres *et al*. also reported a predictive model including 5 variables (cold ischemia time [CIT], recipient age, human leukocyte antigen [HLA] mismatch, panel reactive antibody [PRA], donor age) with an AUC of 0.74 for 1219 recipients [15]. In 2014, Chapal *et al*. proposed a predictive score calculated using 5 parameters (CIT, donor age, recipient Body Mass Index [BMI], donor serum creatinine, anti-thymocyte globulin [ATG] induction) with an AUC of 0.73 [12]. In 2015, Zaza *et al*. have developed a pre-operative predictive model for DGF using 4 variables from the recipient (recipient weight, recipient previous transplant, dialysis way, duration of dialysis) with an AUC of 0.63 for 2,755 patients undergoing deceased donor kidney transplantation [16].

Even though these predictive tools performed well with good discrimination ability to predict DGF risk; however, these models were developed and validated mainly based on patients in the Western countries. The predictive accuracies of these models for Chinese population remain to be investigated. Currently, there is still no DGF predictive model specifically developed for the Chinese population. To identify an appropriate predictive model of DGF risk after deceased donor kidney transplantation for the Chinese population, this study aimed to evaluate the predictive accuracies of the aforementioned five models by external validation using the data of 713 kidney transplantation cases at our center.

## RESULTS

### Demographics and characteristics of donors and recipients

A total of 711 renal transplant cases using deceased donors were included for analysis. According to the status of DGF occurrence, the 711 patients were divided into DGF (*n* = 125) and non-DGF (*n* = 586) groups. The overall incidence of DGF was 17.6%. The demographic and clinical characteristics of donor and recipient were summarized in Table 1. Except for age (*P* < 0.01), there was no significance in the characteristics of recipients between DGF and non-DGF groups (all *P* > 0.05). Donors in DGF group had significantly higher WIT, CIT, terminal serum creatinine, hypertension, cardiac death than those in non-DGF group (all *P* < 0.05). The 1-year, 3-year, 5-year graft survival were 92.6%, 89.0% and 89.0% in DGF group, while were 94.6%, 90.2% and 90.2% in non-DGF group (*p* = 0.498). The 1-year, 3-year, 5-year patient survival were 96.8%, 94.5% and 94.5% in DGF group, while were 97.1%, 94.9% and 94.9% in non-DGF group (*p* = 0.946).

**Table 1:** Demographic and clinical characteristics of donors and recipients at time of transplantation

Characteristics	Total (*n* = 711)	DGF (*n* = 125)	non-DGF (*n* = 586)	*P*
**Recipient**				
**Age (years)**	42 (32–52)	38 (29–48)^*^	43 (33–53)	0.003
**Weight (kg)**	60 (50–65)	59 (50–66)	60 (50–65)	0.388
**BMI (kg/m^2^)**	21.1 (18.6–23.8)	21.3 (18.7–24.5)	21.0 (18.6–23.7)	0.618
**Gender (%male)**	467 (65.7)	84 (67.2)	383 (65.4)	0.411
**Single Organ Transplantation**	702 (98.7)	125 (100.0)	577 (98.5)	0.373
**Previous Transplants (%)**	21 (3)	1 (0.8)	20 (3.4)	0.150
**Diabetes (%)**	94 (13.2)	12 (9.6)	82 (14.0)	0.188
**Transfusion (%)**	135 (19)	22 (17.6)	113 (19.2)	0.628
**Duration of Dialysis (days)**	360 (120–752.5)	360 (118.5–752)	360 (120–752.75)	0.558
**PD (%)**	174 (24.5)	38 (30.4)	135 (23.0)	0.067
**HLA mismatch**	4 (3–4)	4 (4–4)	4 (3–4)	0.126
**ATG induction (%)**	654 (92.0)	116 (92.8)	536 (91.8)	0.667
**Donor**				
**Age (years)**	28 (18–41)	33 (19–42)	28 (18–41)	0.324
**Weight (kg)**	60 (50–70)	61.5 (45–70)	60 (50–69)	0.291
**WIT (minutes)**	0 (0–7)	4.5 (0–15)^*^	0 (0–7)	< 0.001
**CIT (hours)**	10 (6.8–15.5)	11.9 (8–24)^*^	9.6 (6.6–14.3)	0.003
**Terminal Scr (mg/dL)**	1.0 (0.7–1.5)	1.22 (0.92–2.23)^*^	1.0 (0.6–1.4)	< 0.001
**Hypertension (%)**	89 (12.5)	20 (16)^*^	69 (11.8)	0.034
**Cardiac death (%)**	316 (44.4)	85 (68.0)^*^	231 (39.4)	< 0.001
**Anoxia (%)**	43 (6.0)	7 (5.6)	36 (6.1)	0.817
**Cerebrovascular death (%)**	134 (18.8)	18 (14.4)	116 (19.8)	0.161
**Expanded Criteria Donor1 (%)**	39 (5.5)	11 (8.8)	28 (4.8)	0.070
**DGF (%)**	125 (17.6)			

DGF, delayed graft function; BMI, body mass index; PD, peritoneal dialysis; HLA, human leukocyte antigen; ATG, anti-thymocyte globulin; WIT, warm ischemia time; CIT, cold ischemia time.^1^Defined as all donors older than 60 years of age or donor older than 50 years with any two of the following conditions: (1) history of hypertension; (2) terminal serum creatinine > 1.5mg/dL; and (3) cerebrovascular cause of brain death.

### The association between the observed DGF incidences and the calculated DGF risks

The number of patients included for validation of the five models was shown in the Supplementary Table 2. Univariate logistic regression was used to analyze the relations between the observed DGF incidences and the calculated DGF risks. Except for Jeldres 2009 model, there were significant associations between the observed DGF incidence and the calculated DGF risks in the other four predictive models (all *P* < 0.01, Table 2). Among them, Irish 2010 model had the lowest -2 log-likelihood value and the highest Nagelkerke R^2^ value among the five models (Table 2), suggesting that the calculated DGF risk from Irish 2010 model was most associated with the observed DGF incidence among the five models.

**Table 2:** The relationship between observed DGF incidence and calculated DGF risk analyzed by univariate logistic regression

Model	*P* value	OR (95% CI)	-2LL	Nagelkerke R^2^
Irish 2010 (per 1% increase)	< 0.001	1.054 (1.039–1.069)	487.000	0.162
Irish 2010 (≥ 20% vs < 20%)	< 0.001	4.634 (2.969–7.233)	501.818	0.125
Irish 2003 (per 1% increase)	< 0.001	1.041 (1.028–1.053)	507.422	0.119
Chaphal 2014 (per 1% increase)	< 0.001	1.049 (1.030–1.068)	510.390	0.074
Zaza 2015 (per 1% increase)	< 0.01	1.025 (1.008–1.042)	584.513	0.021
Jeldres 2009 (per 1% increase)	0.684	1.005 (0.980–1.031)	541.239	< 0.001

OR, odds ratio; CI, confidence interval; LL, log likelihood.

### Assessment of discrimination ability

**Figure 1:**
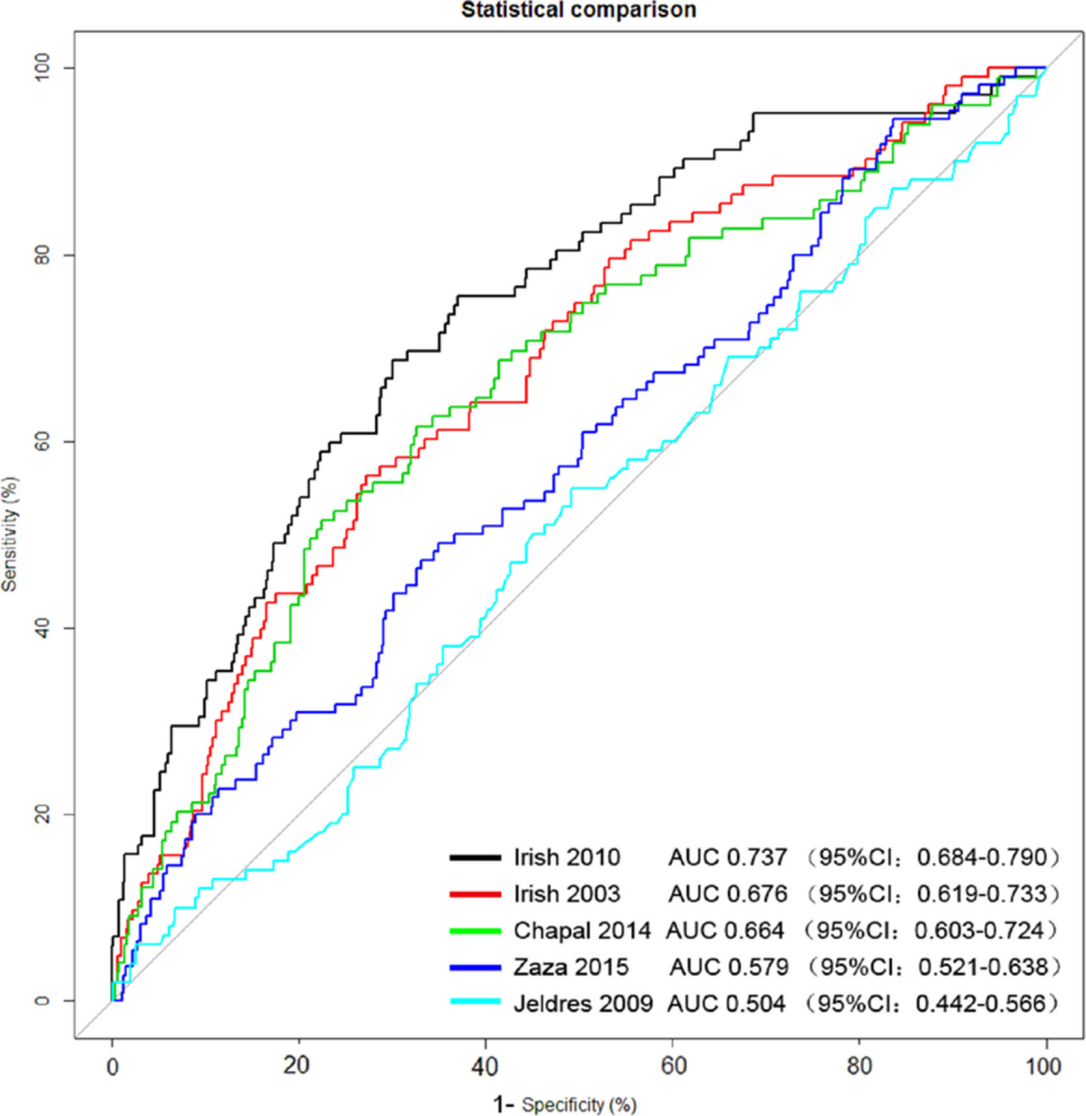
Evaluation of the predictive power of the five models by receiver operating characteristic (ROC) curve using the observed DGF incidence as the standard AUC, area under the ROC curve; CI, confidence interval.

The discrimination ability of the five models was assessed by ROC curve using the observed DGF incidence as the standard. As shown in Figure 1, Irish 2010 model consistently had the highest area under ROC curve, (AUC = 0.737, 95% confidence interval = 0.684–0.790) among the five predictive models, indicating a fair predictive power of Irish 2010 model on the patients of this study. The AUC values for other four models were all small than 0.6, suggesting a poor predictive power.

### Assessment of calibration

**Figure 2:**
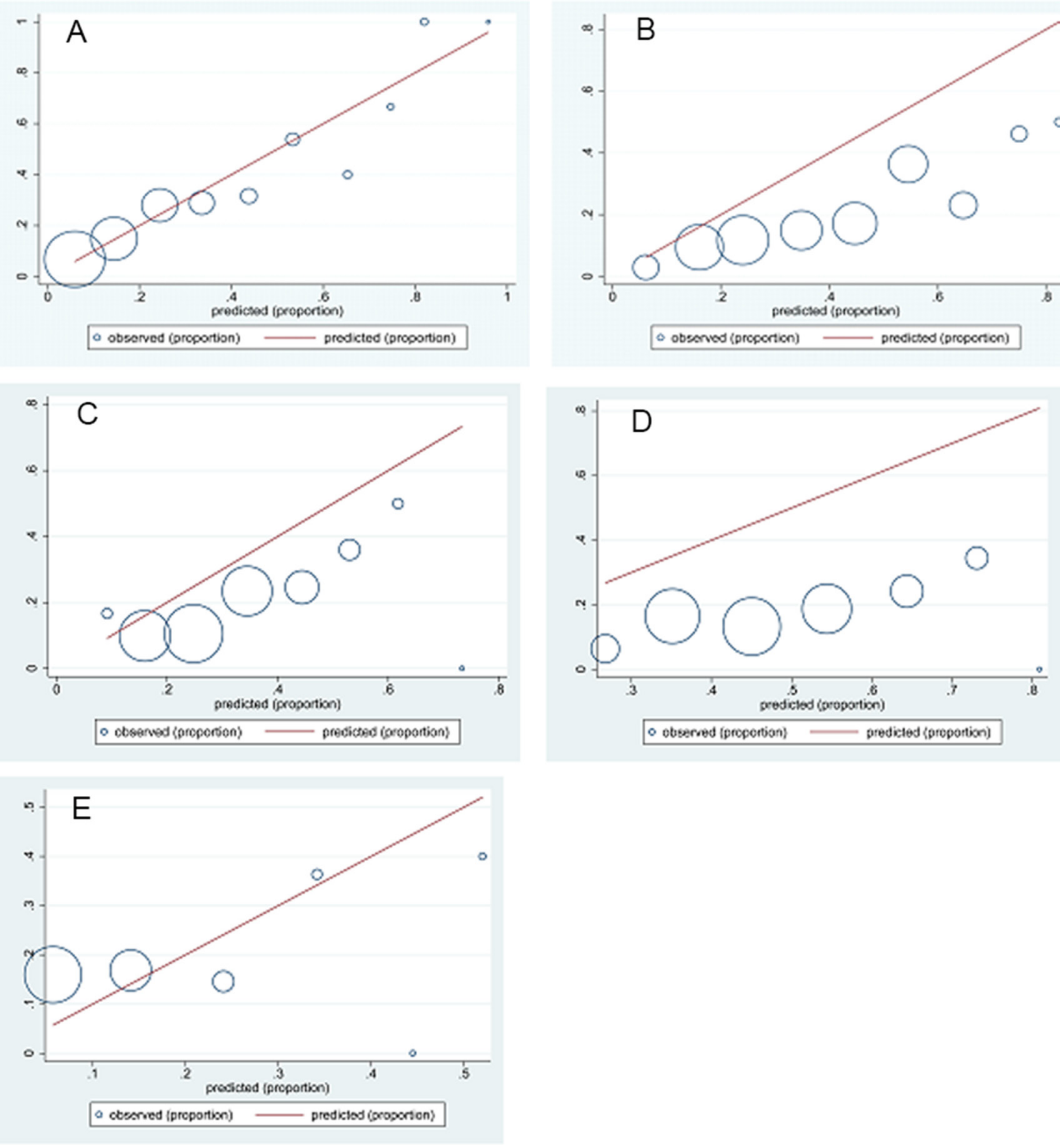
Evaluation of the model calibration by the Hosmer-Lemeshow goodness-of-fit test for Irish 2010 model (**A**), Irish 2003 model (**B**), Chaphal 2014 model (**C**), Zaza 2015 model (**D**) and Jeldres 2009 model (**E**). The observed and predicted delayed graft function (DGF) probabilities were computed according to 10 delayed graft function score (DGF) with intervals at 0.1. The goodness of the fit for Irish 2010 model (A) cannot be rejected (*P* = 0.887, Hosmer–Lemeshow statistic). Both the goodness of the fit for Irish 2003 model (B), Chaphal 2014 (C), Zaza 2015 (D) and Jeldres 2009 (E) were rejected (*P* < 0.05, Hosmer–Lemeshow statistic).

The calibration of models was evaluated by Hosmer-Lemeshow goodness-of-fit test. As shown in the Figure 2, the Irish 2010 model had a perfect calibration between the calculated DGF risk and the observed DGF incidence (*p* = 0.887, Figure 2A), whereas the other four models had poor calibration (both *p* < 0.05, Figure 2B–2E).

### Subgroup analyses

To preliminarily evaluate the predictive accuracy, the calculated DGF risks from the five models were compared between DGF and non-DGF patients in several subgroup analyses including DBD, DCD, ECD, adult donor, child donor, donor aged 5–18 and donor aged ≤ 5. The results showed that in the subgroup analyses of DBD, adult donor and child donor, the calculated DGF risk was significantly different between DGF and non-DGF patients in the four models (Irish 2010, Irish 2003, Chaphal 2014, Zaza 2015, all *P* < 0.05, Table 3). In the subgroup analysis of donor aged 5–18, the significance was observed in the three models (all *P* < 0.05). There were only two and one significances in the DCD and donor aged ≤ 5 subgroups analyses, respectively. Notably, except for ECD subgroup analysis, Irish 2010 model achieved significance in all subgroup analyses. However, we found that in the subgroup of donor aged ≤ 5, there was a large gap between the observed incidence and the calculated risk of DGF by Irish 2010 model (27% vs 9%).

**Table 3:** Comparisons of calculated DGF risks from the five models between DGF and non-DGF patients in several subgroup analyses

Subgroup	Observed incidence of DGF (95% CI)	Model	Total included cases	Calculated DGF risk (median)			*P* value
				DGF group	Non-DGF group	All	
**DBD**	0.10(0.07–0.13)	Irish 2010	372	0.12	0.07	0.07	< 0.001^*^
		Irish 2003	372	0.31	0.22	0.23	0.001^*^
		Chaphal 2014	371	0.34	0.25	0.27	< 0.001^*^
		Zaza 2015	380	0.51	0.44	0.44	0.03^*^
		Jeldres 2009	371	0.08	0.09	0.09	0.94
**DCD**	0.28(0.22–0.34)	Irish 2010	232	0.28	0.21	0.23	< 0.001^*^
		Irish 2003	233	0.52	0.47	0.48	0.12
		Chaphal 2014	228	0.35	0.28	0.31	0.01^*^
		Zaza 2015	275	0.47	0.47	0.47	0.28
		Jeldres 2009	231	0.09	0.08	0.08	0.68
**ECD**	0.28(0.13–0.43)	Irish 2010	39	0.23	0.19	0.21	0.12
		Irish 2003	39	0.44	0.47	0.46	0.96
		Chaphal 2014	39	0.36	0.35	0.38	0.68
		Zaza 2015	39	0.45	0.49	0.48	0.43
		Jeldres 2009	39	0.14	0.18	0.18	0.40
**Adult donor (≥ 18 year old)**	0.18(0.15–0.22)	Irish 2010	481	0.24	0.11	0.12	< 0.001^*^
		Irish 2003	482	0.47	0.30	0.33	< 0.001^*^
		Chaphal 2014	479	0.35	0.27	0.30	< 0.001^*^
		Zaza 2015	501	0.46	0.44	0.44	0.02^*^
		Jeldres 2009	480	0.10	0.10	0.10	0.80
**Child donor (< 18 year old)**	0.14(0.08–0.20)	Irish 2010	123	0.22	0.08	0.09	< 0.001^*^
		Irish 2003	123	0.43	0.23	0.25	0.04^*^
		Chaphal 2014	120	0.28	0.19	0.23	0.04^*^
		Zaza 2015	125	0.52	0.47	0.48	0.04^*^
		Jeldres 2009	122	0.03	0.04	0.04	0.12
**Donor aged 5–18**	0.10(0.04–0.16)	Irish 2010	100	0.26	0.08	0.09	0.01^*^
		Irish 2003	100	0.45	0.27	0.28	0.03^*^
		Chaphal 2014	99	0.33	0.22	0.26	0.02^*^
		Zaza 2015	101	0.49	0.43	0.43	0.33
		Jeldres 2009	99	0.04	0.04	0.04	0.56
**Donor aged ≤ 5**	0.27(0.12–0.39)	Irish 2010	40	0.21	0.06	0.09	0.01^*^
		Irish 2003	40	0.32	0.17	0.18	0.20
		Chaphal 2014	38	0.21	0.17	0.17	0.53
		Zaza 2015	41	0.53	0.53	0.53	0.34
		Jeldres 2009	40	0.02	0.02	0.02	0.86

DGF, delayed graft function; CI, confidence interval; DBD, donation after brain death; DCD, donation after cardiac death; ECD, expanded criteria donors. ^*^Significance in the calculated DGF risk between DGF and non-DGF patient

### Cut-off values determination- clinical usefulness

Hence, different cut-off values (0.1–0.5) were applied to the ROC curve of Irish 2010 model, and the relevant parameters were shown in Table 4. The optimal cut-off values were selected based on the maximum Youden index, defined as the difference between the sensitivity and 1-specificity on the ROC curve [17]. According to the maximum Youden index (36.08%), the optimal cut-off value was 0.2, with a sensitivity of 60.78% and a specificity of 75.3% (Table 4). At the cut-off of 0.1, the model had the best sensitivity (82.35%), negative predictive value (93.08%) and negative likelihood ratio (0.37), while at the cut-off of 0.5, the model had the best specificity (98.01%), positive predictive value (61.54%) and positive likelihood ratio (7.88, Table 4).

**Table 4:** ROC curve analysis of Irish 2010 model with different cut-offs

Cut-off	Sensitivity	Specificity	Positive predictive value	Negative predictive value	Positive likelihood ratio	Negative likelihood ratio	Youden index
0.1	82.35%	48.21%	24.42%	93.08%	1.59	0.37	30.56%
0.2	60.78%	75.30%	33.33%	90.43%	2.46	0.52	36.08%
0.3	36.27%	88.45%	38.95%	87.23%	3.14	0.72	24.72%
0.4	22.55%	95.42%	50.00%	85.84%	4.92	0.81	17.97%
0.5	15.69%	98.01%	61.54%	85.12%	7.87	0.86	13.69%

## DISCUSSION

This is the first study evaluating the predictive models for DGF after deceased kidney transplantation for the Chinese population. In this study, we evaluated the predictive power of five available models. Univariate logistic regression showed that the calculated DGF risk from Irish 2010 model was most associated with the observed DGF incidence among the five models. Irish 2010 model had an AUC of 0.737. Hosmer-Lemeshow goodness-of-fit test showed that the Irish 2010 model had a perfect calibration between the calculated DGF risk and the observed DGF incidence (*p* = 0.887). The calculated DGF risk from Irish 2010 model was significantly different between DGF and non-DGF patients in the subgroup analyses of DBD, DCD, adult donor, child donor, donor aged 5–18 and donor aged ≤ 5, but not ECD. Taken together, these data suggested that Irish 2010 model has the best predictive power among the five models.

In our population, there were significant differences in the recipient age, WIT, CIT, donor terminal serum creatinine, donor history of hypertension and donation after cardiac death between DGF and non-DGF group, most of which was consistent with findings of previous reports [8, 18]. The recipient age was younger in the DGF group as compared with the non-DGF group (38 [29–48] vs. 43 [33–53], *P* = 0.003). The difference in recipient age between DGF group and non-DGF group in our cohort may be attributed to the integrative factors in clinical practice, including organ allocation, surgical decision and patient's willingness. However, among four out of the five models (Irish 2010, Irish 2003, Chaphal 2014, Zaza 2015, all of them with a large sample size) validated in this study, recipient age is not a predictor of DGF, hence there is no age bias in the external validation of these four models. In Jeldres 2009 model (*N* = 532), age > 43 years increases the risk of DGF. In our validation cohort, the proportion of recipients aged > 43 was lower in the DGF group than in the non-DGF group (38.4% *vs*. 51.5%, *p* = 0.008). The lower proportion of recipients aged > 43 in our validation cohort did underestimate the calculated risk in DGF group. Nevertheless, the absolute difference in the proportion of recipients aged > 43 between DGF group and non-DGF group in our cohort was relatively small (13.1%). In addition, among all the included parameters in the Jeldres 2009 model, the recipient age has the least effect on the DGF risk [15]. Therefore, we believed that the age bias in our validation cohort would not alter the conclusion that the discrimination and calibration ability of Jeldres 2009 model was poor for the Chinese population.

The Irish 2010 model has good discrimination and perfect calibration for the Chinese population. Several reasons may contribute to its good predictive power. First, the Irish 2010 model was refined from the Irish 2003 model and made improvements in more risk factors (includes 17 conventional predictors) such as WIT, and more specific inclusion criteria. In addition, Irish 2010 model was developed using the data from the United Network for Organ Sharing (UNOS), which is the largest database among the five models and includes different races, both reduce the selection bias in the logistic regression model. Furthermore, this model has been externally validated using the UNOS 2007 validation dataset as well as Spanish and Belgian population dataset [13, 19, 20].

It is worth mentioning there are some differences in defining and detecting the clinical variables when we externally validated the Irish 2010 model using the data from our population. For instance, PRA variable in our dataset was recorded as “positive or negative PRA” of PRA rather than “percentage of PRA”. Since the mean of PRA in Irish 2010 model was approximately 20%, therefore the variables of positive and negative PRA in our dataset were coded as 20% and 0%, respectively. However, the maximum difference between positive and negative PRA is merely 2 score according to the nomogram of Irish 2010 model, which has little influence on the applicability of the models in China.

To provide references for medical decision making, we compared different cut-offs for the calculated DGF risk from the Irish 2010 model. The first selection method for an appropriate cut-off value is based on the maximum Youden index. At the optimal cut-off of 0.2, the model has the best discrimination. The second method is based on the positive and negative likelihood ratios. At the cut-off of 0.1, the model has the best sensitivity and negative likelihood ratio, while at the cut-off of 0.5, the model has the best specificity and positive likelihood ratio. We believe that these cut-offs would be better because they are less associated with the baseline level of DGF incidence and of more practical value in the clinic. For patients with a calculated DGF risk less than the lower cut-off (i.e. 0.1), DGF could be excluded. For patients with a calculated DGF risk higher than the upper cut-off (i.e. 0.5), appropriate preventive managements or pretreatments should be conducted to prevent the occurrence of DGF and avoid deleterious consequences on long-term graft outcome.

In this study, the overall incidence of DGF was 17.6%, and the DGF rates for DBD and DCD cases were 10% and 28%, respectively. The incidences of DGF in the current study are in line with several recent reports of Chinese population from the other large transplant centers [21–23] and the donor and recipient characteristics are similar between these large transplant centers. These evidences indicated the study population in our center was representative of the national population. The incidence of DGF is lower in our study than in the USA (e.g. Irish *et al*.'s 2010 study, DBD: 25.5%; DCD: 48.2% [13]). One explanation is that the donors in our study were younger; the cause of death was mainly car accident, and the proportion of cerebrovascular accident death was low (18.8%). While in the USA, the proportion of cerebrovascular accident death is usually higher than 40%, and *exhibits a gradually rising trend* [24]. In addition, the donor in our study had a lower incidence of hypertension than those in Irish *et al*.'s 2010 study (12.5% vs 22.4%). Furthermore, the warm ischemic time and cold ischemic time in our study were both shorter than those in Irish *et al*.'s 2010 study (WIT = 39.3 ± 17.6 min; CIT = 17.8 ± 7.8 h [13]). The above reasons contribute to a low risk of DGF in our study cohort.

Although the Irish 2010 model has the best performance for our population among the five models, it still has several limitations. The Irish 2010 model has an AUC of 0.737 for our population, suggesting there is still room for improvement. To improve the discrimination performance, additional risk factors may be considered to be included in the model. For example, ATG induction [8, 25], recipient's residual renal function and perioperative saline loading have been reported be associated with DGF [26], which could be considered to update the Irish 2010 model for the Chinese population. In the several subgroup analyses of this study, the calculated DGF risk between DGF and non-DGF patients reached significance in all subgroup analyses except for ECD, which may be attributed to the small sample size of ECD subgroup analysis (*n* = 39). In the subgroup of donor aged ≤5, the observed DGF incidence is up to 27%, whereas the calculated DGF risk by Irish 2010 model is only 9%. One possible explanation for this difference may be that among our subgroup of donor aged ≤ 5, there were about 40% (15 cases) of donors weighing ≤ 10 kg, and their high incidence of postoperative vascular and ureteral complications would lead to a high risk of DGF [27]. While in the Irish 2010 model, the proportion of donor weighed ≤10 kg is extremely small (0.34% of all deceased donor) [28], therefore this issue could not be considered in the model. However, we cannot conduct further subgroup analysis within the subgroup of donor aged ≤5 due to its small size (*n* = 40). Therefore a study with a large sample size is necessary to validate this issue and to figure out an appropriate predictive model for this subgroup of recipients. It should also be noted that we used the data from a single center in China, and the sample size is still relatively small. It has been suggested that external validation of a prognostic model requires a minimum of 100 events [29, 30], which is much smaller than that for model development (rule of thumb: 5–20 events per variable) [31]. In our validation cohort, 125 events (DGF) were included, thus it was sufficient for unbiased estimation of performance measures of the five models. However, a multicenter study with large sample size should be performed to validate our findings. All these limitations should be addressed in the following study.

## MATERIALS AND METHODS

### Study population

To evaluate the predictive power of the five models (Irish 2010 [13], Irish 2003 [14], Chaphal 2014 [12], Zaza 2015 [16], Jeldres 2009 [15]) for the DGF risk after deceased donor kidney transplantation for Chinese population, the medical records of 713 patients receiving solitary renal transplantation using deceased donors from organ donation after brain death (DBD) and circulatory death (DCD) [32] in the First Affiliated Hospital, Sun Yat-sen University between February 2007 and August 2016 were retrospectively reviewed. No organs from executed prisoners were used. Two patients without the record of DGF status were excluded from the analysis. In addition, the patients were screened for the validation cohort of the five models according to their own inclusion criteria (Supplementary Table 1, Supplementary Figure 1). In the analysis for the Irish 2010 model and Irish 2003 model, the recipients aging < 16 years were excluded, while the recipients aging < 18 years were excluded in the other models. This study was approved by the institutional review board of the First Affiliated Hospital of Sun Yat-sen University.

### Data collection

Data including demographic and pre-/post-transplant-related clinical characteristics of donors and recipients, follow-up events and outcomes were collected. DGF was defined as the need for dialysis within the first week post-transplant [2]. The five predictive models were presented in different formats including regression formula, nomogram, web-based calculator and scoring system (Supplementary Table 2). The DGF risk was mainly calculated from the regression formula in the model. If the regression formula was not available in the model, the DGF risk was calculated for each recipient using nomogram, web-based calculator or scoring system. The higher the calculated DGF risk is, the more likelihood that DGF develops in the patient. The variables included for analysis of the five predictive models were listed in Supplementary Table 2.We had complete data for most of the variables; while there was some missing data (< 5–10%, except for PRA) needed imputation. They were missing at random. Calculated PRA (cPRA) was not available in China. Therefore, negative and positive PRA in our dataset were coded as 0% and 20% (median of cPRA in Irish 2010 model). Missing duration of dialysis was recorded as 360 days (median of available data). Missing HLA mismatches was recorded as 4 (median of available mismatch number). Missing donor terminal serum creatinine was considered normal and estimated with age and sex according to the modification of diet in renal disease (MDRD) equation (> 16 years old) [33] or Schwartz equation (≤ 16 years old) (Normal GFR = 90 ml/min/1.73m^2^) [34]. Missing donor weight was estimated with age and sex according to data from General Administration of Sport [35]. For categorical data, missing or unknown were grouped into the “no” or “absence” category.

### Statistical analysis

Continuous data with normal distribution was presented as the mean ± standard deviation (SD), while continuous data without normal distribution was expressed as median (lower quartile-higher quartile). Categorical data was reported as counts and percentages. Kaplan-Meier method was used for survival analysis and the log-rank test was used to compare two survival curves. The relations between the observed incidence of DGF and the calculated DGF risk were analyzed using univariate logistic regression. Nagelkerke R^2^ and -2Log Likelihood were used for the evaluation of the regression model performance. The predictive accuracy of the five models on our data was assessed using the concordance index (AUC), which estimates the probability of concordance between predicted and observed responses. The cut-off value was determined based on the Youden index as well as a positive/negative likelihood ratio. The Hosmer-Lemeshow “goodness-of-fit” test was taken for model calibration [36]. The calculated DGF risks from the five models were compared between DGF and non-DGF patients using Mann-Whitney U test. Two-group data comparison, ROC curve and logistic regression model were analyzed using IBM SPSS Statistics version 22.0 (IBM Corporation, New York, USA). Hosmer-Lemeshow “goodness-of-fit” test was performed with Stata MP version 14.0 (STATA Corporation, Texas, USA). Model calibration was performed using ‘R’, a free software environment for statistical computing and graphics (www.r-project.org).

## CONCLUSIONS

In summary, our study shows that Irish 2010 model has good predictive power for DGF risk after deceased donor kidney transplantation in the Chinese population, which may be helpful in optimizing organ utilization decision and guiding preventive and therapeutic strategies.

## SUPPLEMENTARY MATERIALS FIGURE AND TABLES



## Abbreviations

DGF: Delayed graft function
ECD: expanded criteria donors
DCD: donation after cardiac death
CIT: cold ischemia time
HLA: human leukocyte antigen
PRA: panel reactive antibody
DBD: donation after brain death
DCD: donation after circulatory death
MDRD: modification of diet in renal disease
AUC: assessed using the concordance index
UNOS: United Network for Organ Sharing
