# Supplementary material for: Evaluation of predictive models for delayed graft function of deceased kidney transplantation
Source: Oncotarget. 2017 Nov 27;9(2):1735–44. doi: 10.18632/oncotarget.22711 (PMC5788595; doi:10.18632/oncotarget.22711)
Supplement: Supplementary file 1 [file oncotarget-09-1735-s001.pdf]

# Evaluation of predictive models for delayed graft function of deceased kidney transplantation

## SUPPLEMENTARY MATERIALS

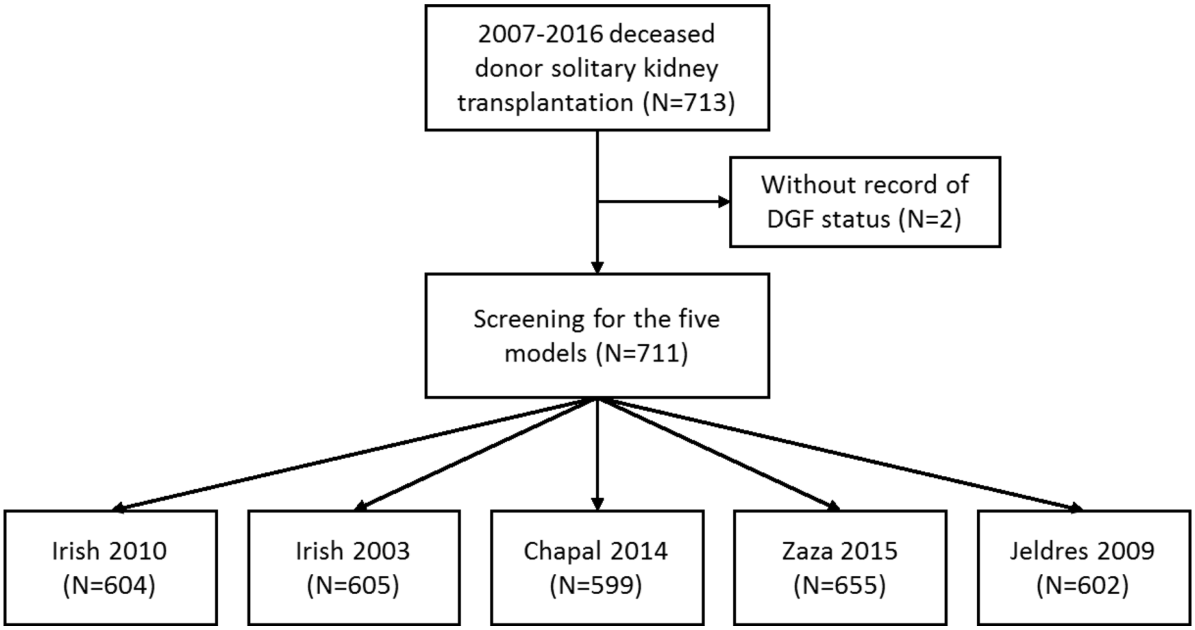


**Supplementary Figure 1: Flow chart of patient selection.**

**Supplementary Table 1: Selection criteria and number of patients selected for validation of the five models**

| Model | Inclusion criteria | Total included cases |
|---|---|---|
| Irish 2010 | 1) Nonpreemptive, nonmachineperfused<br>2) Adult (≥ 16 years) recipients<br>3) Recipient's BMI, CIT, WIT, donor sex and donor age data available | 604 |
| Irish 2003 | 1) Adult (≥ 16 years) recipients<br>2) CIT, donor sex and donor age data available | 605 |
| Chapal 2014 | 1) Non pre-emptive, nonmachine-perfused<br>2) Adult (≥ 18 years) recipients<br>3) Recipient's BMI, CIT, donor sex and donor age data available | 599 |
| Zaza 2015 | 1) Adult (≥ 18 years) recipients<br>2) Recipient's body weight data available | 655 |
| Jeldres 2009 | 1) Adult (≥ 18 years) recipients<br>2) CIT and donor age data available | 602 |

CIT: Cold ischemic time, WIT: Warm ischemic time.

**Supplementary Table 2: Sample variables and presentation format of the five predictive models for DGF after deceased donor kidney transplantation**

| Model | Samples for analysis in the original model | Sample size of original models | Variables | Variable count | Presentation format |
|---|---|---|---|---|---|
| Irish 2010 | United Network for Organ Sharing/Organ Procurement and Transplantation (UNOS/OPTN) on adult (≥16 years) recipients of a solitary, nonpreemptive, nonmachineperfused, deceased donor kidney between January 1, 2003 and December 31, 2006 | 24 337 | peak panel reactive antibodies(PRAs), dialysis duration, recipient body mass index (BMI), cold ischemia time (CIT), warm ischemia time, recipient race, recipient sex, recipient previous transplant, diabetic recipient, pre-transplant transfusion, human leukocyte antigen (HLA) mismatch, donor age, donor weight, nonheart-beating donor, donor hypertension, donor cause of death, and donor serum creatinine | 17 | Nomogram, web-based risk calculator (www.transplantcalculator.com/DGF) |
| Irish 2003 | Adult (aged ≥16 yr) recipients of cadaveric renal allografts over a 4-yr transplantation period (1995 to 1998) were obtained from the USRDS registry | 13,846 | PRA, pre-transplant dialysis, single organ transplant, CIT, recipient race, recipient sex, recipient previous transplant, diabetic recipient, pre-transplant transfusion, HLA mismatch, donor age, donor weight, nonheart-beating donor, donor hypertension, donor cause of death, and donor serum creatinine | 16 | Nomogram |
| Chapal 2014 | Adult (≥18 years) recipients of isolated, non pre-emptive, nonmachine-perfused, deceased donor kidneys, who were prospectively collected since January 2007 and computerized in the DIVAT | 1219 | CIT, donor age, recipient BMI, donor serum creatinine, anti-thymocyte globulin (ATG) induction | 5 | Regression formula, DGFS scoring system |
| Zaza 2015 | Adult (≥18 years) that has been compiled by four renal/transplant units in Italy from 1984 to 2012. | 2755 | Recipient weight, recipient previous transplant, dialysis way, duration of dialysis | 4 | Regression formula |
| Jeldres 2009 | Adult (≥18 years) underwent RT from deceased donors at the University of Montréal Health Centre between 1979 and 2004 | 532 | CIT, recipient age, HLA mismatch, PRA, donor age | 5 | Nomogram |
